# Management of Post-Operative Crohn’s Disease: Knowns and Unknowns

**DOI:** 10.3390/jcm13082300

**Published:** 2024-04-16

**Authors:** Matteo Spertino, Roberto Gabbiadini, Arianna Dal Buono, Anita Busacca, Gianluca Franchellucci, Giulia Migliorisi, Alessandro Repici, Antonino Spinelli, Cristina Bezzio, Alessandro Armuzzi

**Affiliations:** 1 Department of Biomedical Sciences, Humanitas University, Via Rita Levi Montalcini 4, Pieve Emanuele, 20072 Milan, Italy; matteo.spertino@humanitas.it (M.S.); gianluca.franchellucci@humanitas.it (G.F.); giulia.migliorisi@humanitas.it (G.M.); alessandro.repici@hunimed.eu (A.R.); antonino.spinelli@hunimed.eu (A.S.); cristina.bezzio@hunimed.eu (C.B.); 2 IBD Center, Department of Gastroenterology, IRCCS Humanitas Research Hospital, Via Manzoni 56, Rozzano, 20089 Milan, Italy; roberto.gabbiadini@humanitas.it (R.G.); arianna.dalbuono@humanitas.it (A.D.B.); anita.busacca@humanitas.it (A.B.); 3 Endoscopy Unit, Department of Gastroenterology, IRCCS Humanitas Research Hospital, Via Manzoni 56, Rozzano, 20089 Milan, Italy; 4 Division of Colon and Rectal Surgery, IRCCS Humanitas Research Hospital, Via Manzoni 56, Rozzano, 20089 Milan, Italy

**Keywords:** Crohn’s disease, ileo-colonic resection, post-operative recurrence, prophylaxis strategy, risk factors

## Abstract

Crohn’s disease (CD) is a chronic inflammatory disorder of the gastrointestinal tract characterized by relapsing–remission phases. CD often requires surgical intervention during its course, mainly ileo-cecal/ileo-colonic resection. However, surgery in CD is not curative and post-operative recurrence (POR) can happen. The management of CD after surgery presents challenges. Ensuring timely, effective, and safe therapy to prevent POR is essential but difficult, considering that approximately 20–30% of subjects may not experience endoscopic POR and that 40–50% will only exhibit intermediate lesions, which carry a low risk of mid- and long-term clinical and surgical POR. Currently, there are two accepted intervention strategies: early post-operative prophylactic therapy (systematically or based on the patient’s risk of recurrence) or starting therapy after confirming endoscopic POR 6–12 months after surgery (endoscopy-driven prophylactic therapy). The risk of overtreatment lies in exposing patients to undesired adverse events, along with the costs associated with medications. Conversely, undertreatment may lead to missed opportunities to prevent bowel damage and the necessity for additional surgery. This article aims to perform a comprehensive review regarding the optimal strategy to reduce the risk of POR in CD patients and the current therapeutic options.

## 1. Introduction

Crohn’s disease (CD) is a life-long intestinal disorder characterized by a chronic relapsing inflammation of the gastrointestinal tract, with a continuously increasing incidence [1]. The pathogenesis of CD is not yet fully understood. Presumably, it is an immune-mediated disease that originates from an abnormal immune response to the gut microbiota in genetically susceptible hosts [2,3].

The inflammation is typically transmural and can eventually lead to fibrosis and luminal narrowing resulting in a bowel obstruction, or it can also develop fistulous tracts and abscesses [4].

Due to its stenotic and fistulizing behavior, CD often requires surgical intervention during its course, with rates of approximately 50% in the 20 years following the diagnosis [5].

Indeed, despite the established effectiveness of biological therapy, which has reduced the risk of surgery over time, the rate of patients needing surgery still remains high [6].

The primary surgical procedure is ileo-cecal/ileo-colonic resection (ICR), as CD typically localizes along the last portion of the small intestine and the first segment of the large intestine [7].

However, unlike ulcerative colitis, surgery in CD is not curative, and there is a risk of post-operative recurrence (POR), which can occur very early, even a few weeks after the intervention, and most frequently involves the anastomotic site [8].

The pathogenesis of CD recurrence after surgery involves the microbiome, creeping fat, genetics and immune components. Despite numerous studies, the mechanisms through which the abovementioned factors act are still unclear [9].

This exposes the patient to the possibility of multiple surgical interventions during their lifetime, increasing the risk of short bowel syndrome [10].

Hence, there is a need to implement actual knowledge about the optimal measures that can prevent disease recurrence. Currently, there are two accepted intervention strategies: starting post-operative prophylactic therapy (systematically or based on the patient’s risk of recurrence) or starting therapy only after confirming endoscopic POR 6–12 months after the intervention (endoscopy-driven post-operative prophylactic therapy) [11].

Each strategy displays potential advantages and disadvantages, and even international guidelines state heterogeneous conclusions on the best management of POR prophylaxis [12,13,14].

Furthermore, several studies have investigated the efficacy of various drugs for the prevention of POR, with conflicting results, and recently, novel mechanisms of action (i.e., vedolizumab and ustekinumab) have increased the medical options for disease control [15].

This article aims to perform a comprehensive review of the evidence regarding the optimal strategy to reduce the risk of POR in CD patients after ICR and the current therapeutic options.

## 2. Pathophysiology of Post-Operative Recurrence

**Figure 1:**
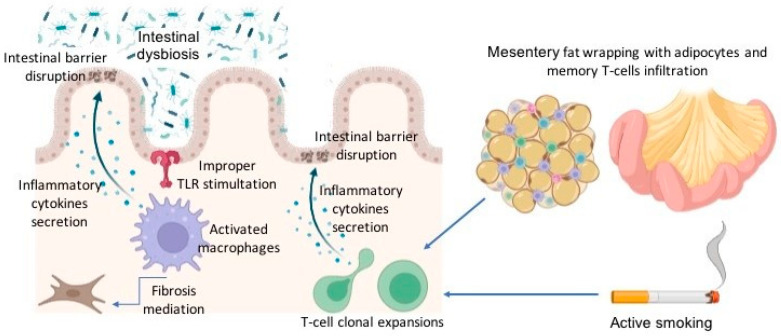
Overview of the main pathophysiological mechanisms behind post-operative recurrence.

The pathophysiology of CD POR is complex and probably more obscure than that of unoperated CD. The main factors thought to be involved in this process are the microbiome, the mesenteric creeping fat, the immune system, and genetic susceptibility [16,17] (Figure 1).

The microbiota plays a significant role in the pathogenesis of POR, as demonstrated by a pivotal study [18] that defined the association between the luminal content and the risk of POR. Stasis of the luminal content at the anastomotic level, particularly in certain types of anastomoses (e.g., side-to-side anastomoses), promotes bacterial proliferation and the subsequent action of some bacterial strains on the ileo-colic mucosa, where the immune system is already primed to trigger the inflammatory response [18].

Similarly, Sokol et al. demonstrated that there is an intense dysbiosis in subject with CD after ileo-cecal resection and that endoscopic recurrence is related to strong differences in the ileal mucosa-associated microbiota compared with non-recurrence (increment of Proteobacteria phylum and reduction in the Firmicutes phylum) [19].

With regard to the genetic component, the identification of the first gene (NOD2) correlated with CD demonstrated the significance of microbial–immune system interplay in the process of the disease, considering the gene’s function in recognizing bacterial peptides. Similarly, a recent meta-analysis observed an association between the presence of NOD2 and the likelihood of POR (cumulative odds ratio: 1.64, *p* = 0.003) [20].

In addition to NOD2, other genes, including BACH2, CARD8, TNFSF15, IRGM, IRF8, LSP1/TNNI2, DAP, PTGER4, PELI3, CHL1, PARVB, and STK24, have been implicated in the POR of CD. It is worth noting that these genes were identified in separate cohort studies, and their discovery lacked a unified biological process or mechanism [20]. As a result, to date, genetic testing should not be a routine aspect of daily clinical practice for identifying individuals at risk of POR [17].

From the perspective of the immune system, it is known that the innate immune system serves as the initial self-defense mechanism. It encompasses physical barriers, such as mucosal cells that can actively contribute through their secretions, along with immune cells like granulocytes, monocytes, and dendritic cells [16].

Macrophages play a pivotal role in the innate immune system, serving as a crucial bridge to acquired immunity, producing pro-inflammatory cytokines (in response to improper TLR stimulation), granuloma formation, mediation of fibrosis, and disruption of the intestinal epithelial barrier function [16,17,21,22]. However, their precise actions in POR remain less understood. Interestingly, in the study by Zorzi et al., it has been observed that prior to the development of visible endoscopic abnormalities, the mucosa in the neo-terminal ileum exhibited a high presence of macrophages, further supporting the concept that macrophages play a primary role in instigating the inflammatory events essential for the POR [23].

Regarding the adaptative immune system, cell-mediated immunity may be the primary player in the recurrence process [16]. T cells have a central role in cell-mediated immunity by detecting and targeting a wide range of antigens and regulating the immune response. T cells accumulate in inflamed tissues and contribute to mucosal injury through their secretion of inflammatory cytokines. An interesting study by Allez et al. showed that the mucosa of the affected bowel of operated CD patients harbors significantly elevated clonal expansions of T cells in comparison to that of healthy individuals [24]. Furthermore, smoking behavior is also associated with an increased proportion of T cell clonal expansions, and these clonal expansions in the mucosa were associated with POR [24].

Lately, there has been a growing interest in the role of the small bowel mesentery in CD recurrences due to findings indicating that the extent of the mesentery removed, along with the affected bowel, has an impact on the POR rate [25].

The mesentery serves as a storage site for inflammatory cells housed within lymph nodes. In CD, the mesentery exhibits a distinctive phenomenon called “fat wrapping” or “creeping fat”, which involves a unique form of adipose tissue hypertrophy. Creeping fat is characterized by numerous small adipocytes accompanied by immune cell infiltration, including regulatory macrophages and memory T cells [9,16].

Hence, it is plausible to regard mesenteric lymph nodes as the repository of CD immune memory, which can be recruited back to the mucosa through homing mechanisms and potentially lead to recurrence [16].

However, the benefit of mesenteric excision has been evaluated in retrospective studies, with contrasting results [25,26,27].

Therefore, robust evidence is lacking and the standard mesenteric excision during ICR is not recommended [17,28].

## 3. Risk Factors for POR

Several risk factors for POR have been identified and they are generally divided into subgroups, such as factors related to the patient, to the disease behavior, surgery type, and histological features [12].

### 3.1. Smoking

Smoking is a well-established risk factor associated with CD post-operative recurrence, either clinical, endoscopic, or surgical [29,30,31,32,33,34].

A meta-analysis, which included 16 studies and 2962 patients (48.1% non-smokers, 47.0% smokers, 4.6% ex-smokers), showed that smokers had significantly higher post-operative clinical recurrence (OR = 2.15; 95% CI 1.42–3.27; *p* < 0.001) and surgical recurrence (OR = 2.56; 95% CI 1.79–3.67; *p* < 0.001) than non-smokers [33].

Similarly, another more recent meta-analysis including 33 studies reported an increased odds of disease flare after ICR (OR 1.97; 95% CI 1.36–2.85) and an increased need to re-undergo surgery (OR 2.17; 95% CI 1.63–2.89) in smokers compared to non-smokers [35].

Furthermore, smoking not only increases the recurrence rate of CD but also aggravates the disease course, causing frequent relapses and a shorter time to recurrence [35,36].

Interestingly, the amount of cigarettes smoked in a day also has an impact on the risk of POR. Indeed, in the study by Lindberg et al., heavy smokers (>10 cigarettes per day) experienced a higher rate of surgical recurrence after 10 years of follow up compared with moderate or non-smokers (26% in never smokers, 28% in smokers ≤ 10 cigarettes per day, 42% in heavy smokers; heavy smokers vs. never smokers OR 1.79, 95% CI 1.12–2.55, *p* = 0.015) [37].

Two other studies reported a higher risk of symptomatic relapse in heavy smokers than in mild smokers [38,39].

Additionally, smoking cessation after ICR may significantly reduce the long-term risk of surgical recurrence and smoking cessation is beneficial at any stage, including in the perioperative setting [11,40,41]. As proof of this fact, also in the above-mentioned meta-analysis, no difference was observed in terms of the surgical POR risk between ex-smokers and non-smokers at 10 years of follow up (OR 0.30; 95% CI 0.09–1.07; *p* = 0.10) [33].

Cigarette smoking is the only modifiable risk factor for POR. Therefore, initiatives should focus on improving communication methods to instruct patients about the broad health risks, including the recurrence of Crohn’s disease after surgery, associated with smoking. Simultaneously, support to quit smoking should be provided through counseling and referral should be made to a smoking discontinuation program [11].

### 3.2. Disease Characteristics

The role of disease characteristics, such as the penetrating phenotype or previous ICR, as potential risk factors for POR is less clear than that of smoking.

In the RCT by McLeod et al., the authors demonstrated that previous resections predicted a higher risk of both endoscopic (OR = 1.78; CI 95% 1.06–2.90; *p* = 0.028) and clinical (OR = 2.0; CI 95% 1.14–3.60; *p* = 0.0016) recurrence [42].

Similarly, in the prospective study of 225 patients conducted by Auzolle et al., previous resection was associated with a higher risk of endoscopic POR (OR = 3.03; CI 95% 1.36–7.12) [30]. A recent observational study by Joustra et al. replicated the same association (OR 2.58; 95% CI 1.07–6.22; *p* = 0.03) [29].

Regarding the penetrating disease, Ozgur et al. retrospectively reported an association between penetrating behavior (*p* = 0.011), intra-abdominal abscess (*p* = 0.040) and post-operative disease recurrence [43]. A subsequent meta-analysis of six RCTs reproduced these results. In the Poisson regression analysis, penetrating disease behavior was associated with endoscopic recurrence (RR 1.27, 95% CI 0.90–1.78) [44].

However, these data are in contrast with the randomized post-operative Crohn’s endoscopic recurrence (POCER) trial, in which previous resection (OR 1.5; *p* = 0.41) and penetrating disease (OR 0.9; *p* = 0.78) were not associated with an increased risk of endoscopic recurrence. Only smoking and the presence of two or more clinical factors, including smoking, increased the risk of ER [32].

Previous resection and penetrating disease also failed to predict recurrence in other studies. In an international multicenter study of 127 patients conducted by de Barcelos et al., penetrating disease behavior (OR 0.98, *p* = 0.99) and previous resection (OR 0.81, *p* = 0.69) were not associated with a risk of endoscopic recurrence [45].

Surprisingly, in the study by Maggiori et al., penetrating behavior was an independent predictor of reduced endoscopic (OR = 0.58; 95% CI 0.39–0.86; *p* = 0.007) and clinical (OR = 0.36; 95% CI 0.16–0.81; *p* = 0.013) recurrence. However, post-operative prophylactic therapy is often prescribed in penetrating CD, representing a management bias [46].

More recently, a large prospective multicenter study (*n* = 365 patients) confirmed the absence of an association between traditional clinical risk factors and endoscopic recurrence. Again, only post-operative smoking was associated with recurrence (OR 2.78, 95% CI 1.16–6.67) [31].

### 3.3. Histological Risk Factors

#### 3.3.1. Granulomas

It has been hypothesized that epithelioid granulomas characterize individuals with a more aggressive disease course, and their role as a risk factor for POR has long been debated [47,48]. A meta-analysis of 2236 patients (21 studies) reported that the number of recurrences and reoperations was significantly higher in subjects with granulomas in the ICR specimens compared to those without granulomas (OR: 1.37; *p* = 0.04; OR: 2.38, *p* < 0.001, respectively). Furthermore, similar to smoking, individuals with granulomas displayed a significantly shorter time to recurrence [49].

A recent meta-analysis comprising 1777 patients confirmed this association: granulomas increased the risk of clinical (RR 1.31; 95% CI 1.05–1.64) and endoscopic (RR 1.37; 95% CI 1.00–1.87) recurrence [50].

Granulomas in the mesenteric lymph nodes (MLNs) may be associated with a higher risk of POR as well. Li et al. exposed that the presence of MLN granulomas was a risk factor for endoscopic POR (HR = 1.91; 95% CI 1.06–3.45; *p* = 0.031) as well as surgical POR (HR = 3.43; 95% CI 1.18–9.99; *p* = 0.023). However, in contrast to previous studies, granulomas in the bowel wall were not associated with endoscopic or surgical recurrence [51].

Unger et al. recently investigated the role of mesenteric granulomas by following 274 patients for a median of 8.54 years. In a multivariate analysis, the presence of mesenteric granulomas significantly influenced the risk of surgical recurrence (HR 1.94; 95% CI 1.04–3.60; *p* = 0.037) [52].

New and better designed studies are needed to clarify the real impact of granulomas in predicting recurrence.

#### 3.3.2. Plexitis

Plexitis is defined as the presence of inflammatory cells contiguous to or within an enteric nerve bundle [17]. The pivotal research on this topic, conducted by Ferrante et al., highlighted that patients with myenteric plexitis (MP) of the proximal resection margin, without other signs of inflammation at the resection margin, had a higher endoscopic POR at 3 months (75% vs. 41%; OR 4.36; 95% CI 1.44–13.23; *p* = 0.008) and at 1 year (93% vs. 59%; OR 9.80; 95% CI 1.04–92.70; *p* = 0.041). In addition, these patients also had a major risk of redo surgery [53]. Subsequently, the retrospective study conducted by Misteli et al. (*n* = 86 patients) also showed the association between severe plexitis and surgical recurrence (*p* = 0.035) [54]. Again, another retrospective study (*n* = 75 patients) observed that the presence of myenteric plexitis in the proximal margins of ICR specimens was independently associated with endoscopic POR (HR 8.83, 95%CI 1.6–48.6, *p* = 0.012) and clinical POR (HR 4.02, 95%CI 1.4–11.2, *p* = 0.008) [55].

Not only myenteric plexitis but also submucosal plexitis has a potential role in POR. A French study demonstrated that submucosal plexitis with ≥three mastocytes was associated with early clinical recurrence [56].

Subsequently, Bressenot et al. suggested that either the presence of at least one eosinophil or more than six lymphocytes in the most severely inflamed ganglion of the submucosal plexus was independently associated with the risk of surgical recurrence [57].

These results were corroborated by a more recent prospective study that showed the submucosal lymphocytic plexitis in the proximal surgical margin was associated with a higher risk of POR (*p* = 0.02) [58].

However, myenteric plexitis can also be present in other intestinal diseases, such as enteric dysmotility, gastroparesis, achalasia, and diverticulitis. The significance of myenteric plexitis in diverticulitis and Crohn’s disease remains unclear. The observation that MP was found in complicated diverticulitis but not in chronic diverticular disease suggests that MP may be a marker of transmural inflammation and more aggressive disease [54,59,60].

#### 3.3.3. Resection Margins

Extended resection with wide margins free from macroscopic inflammation is not associated with a lower rate of recurrence, and today, a bowel-sparing surgery is recommended. In fact, an RCT comprising 152 individuals with CD undergoing ICR randomized the patients into limited resection (proximal line of resection 2 cm from the limit of macroscopically diseased bowel) or extended resection (proximal line of resection 12 cm from the limit of macroscopically diseased bowel). After a median follow-up of 55.7 months, the rate of disease recurrence between the two groups were not significantly different (25.3% in limited resection vs. 17.9% in extended resection, *p* = 0.31) [61].

While this landmark trial cleared up the issue regarding the macroscopic resection margins, the question of microscopic resection margins is not completely answered.

Indeed, in the above-mentioned RCT, the recurrence rates did not increase when microscopic disease was present at the resection margins [61].

Conversely, a meta-analysis of 18 studies comprising 1833 patients showed that histopathological margin positivity was associated with a higher rate of overall recurrence (OR 1.7; 95% CI 1.3–2.1; *p* < 0.001), clinical recurrence (OR 1.7; 95% CI 1.0–2.8; *p* = 0.04) and anastomotic recurrence (OR 1.6; 95% CI 1.0–2.3; *p* = 0.03) [62].

Three subsequent studies and a more recent meta-analysis confirmed these data, also observing a correlation between the microscopic positive margins and early endoscopic POR (at 6 months) [50,63,64,65], suggesting a possible residual disease after surgery due to margin positivity rather than early POR [66].

However, in the prospective multicenter study conducted by Arkenbosch et al., incorporation of histological factors improved the predictive value for endoscopic recurrence of only 3% compared to using clinical risk factors alone [67].

Therefore, even if microscopic positive margins are a potential risk factor for POR, in routine surgical practice bowel-sparing surgery is still the aim and intraoperative frozen sections during ICR are not recommended [17].

### 3.4. Surgical Risk Factors

#### 3.4.1. Anastomotic Techniques

Surgical strategies to reduce POR after ICR have historically focused on the technique and the type of anastomosis. Several studies have investigated anastomotic techniques, in particular the side-to-side anastomosis (SSA) compared to the end-to-end anastomosis (EEA). Most studies were retrospective and included a mix of both stapled and handsewn anastomoses [68,69,70,71,72].

A randomized trial of 170 patients comparing SSA and EEA conducted by McLeod et al. demonstrated that the endoscopic and clinical recurrence rate after a mean follow up of 11.9 months were similar between the two groups (37.9 vs. 42.5%, *p* = 0.55 for endoscopic POR, and 22.7 vs. 21.9%, *p* = 0.92 for clinical POR, respectively) [42].

This is in contrast to an Italian RCT, which found that end-to-end anastomosis had a more than 3-fold higher risk of endoscopic POR than any other type of anastomotic configuration [73].

Guo et al. published a meta-analysis of 11 studies that compared side-to-side anastomosis to other anastomotic techniques (handsewn EEA, handsewn end to side, stapled EEA and handsewn end to side), observing a lower overall rate of post-operative complications (OR 0.60; *p* = 0.01) but no difference in endoscopic recurrence, symptomatic recurrence, and reoperation rates. Only stapled side-to-side anastomosis could reduce the reoperation rates (HR 0.38; *p* = 0.01) [74]. A subsequent meta-analysis by He et al., which included 821 patients from 8 studies, compared handsewn end-to-end anastomosis with stapled side-to-side anastomosis. They found that the rate of overall short-term post-operative complications, the recurrence rate and the need for reoperation were all lower in the stapled side-to-side anastomosis group [75].

Finally, a systematic review and network meta-analysis (1113 patients from 11 studies) revealed the probably SSA was associated with an overall reduction in post-operative complications (OR 0.54, 95% CI 0.34–0.83), clinical recurrence (OR 0.32, 95% CI 0.13–0.77) and redo surgery (OR 0.22, 95% CI 0.05–0.95) [76]. Summing up, the quality of the studies included in all the meta-analyses was low, with a minority of patients included in RCTs.

In general, stapled side-to-side anastomosis seems the favorite technique, but this remains controversial based on the available literature.

About this, two international parallel multicenter RCTs with a similar setup, comparing handsewn (end-to-end and Kono-S) to stapled (side-to-side) anastomosis, will be conducted mainly in the Netherlands (End2End) and Italy (HAND2END), with the objective of determining which type of anastomosis is superior with respect to endoscopic recurrence [77].

#### 3.4.2. Kono-S Anastomosis

A new anastomotic technique called Kono-S anastomosis was described by Kono in 2011. This technique consists of a hybrid manual and stapled anastomosis based on the concept of mesenteric exclusion: the mesentery of the affected intestinal segment is divided at the mesenteric edge of the bowel to avoid devascularization and denervation of the residual bowel, thus preserving the blood supply and neural control. Furthermore, this technique permits obtaining a wide anastomosis without a large blind stump, minimizing the fecal stasis and bacterial overgrowth, which have a role in the pathogenesis of recurrence [78].

Kono et al. revealed a lower endoscopic recurrence score in patients who underwent Kono-S anastomosis compared with patients who underwent conventional anastomosis (*p* = 0.008) [78]. In the first RCT regarding this topic, 36 patients were randomized to the Kono-S anastomosis and 43 patients to stapled SSA. The endoscopic recurrence rate at 6 months was lower in the Kono-S group (22.2 vs. 62.8%, *p* < 0.001, respectively). Clinical recurrence was similar between the two groups at 12 months (8 vs. 18%, *p* = 0.2); however, it was lower in the Kono-S group at 24 months (18 vs. 30.2%, *p* = 0.04). The surgical recurrence rates at 24 months were similar between the two groups (0 vs. 4.6%, *p* = 0.3) [79]. Two meta-analyses confirmed the effectiveness of Kono-S in preventing endoscopic and surgical recurrence when compared to other types of anastomoses, and they also confirmed the safety of this technique [80,81].

Conflicting data arose from a recent prospective multicenter RCT (results published in the form of an abstract) comparing the Kono-S anastomosis to side-to-side anastomosis. A total of 288 patients were enrolled: 154 patients were randomized to the Kono-S group and 134 to the side-to-side group. Three to six months after ICR, there was no significant difference between the two groups in terms of clinical (*p* = 0.109) and endoscopic POR (*p* = 0.883) or in recurrence-free survival (*p* = 0.256). It should be highlighted that in the Kono-S group, there was a higher percentage of past smokers (57% vs. 30%, *p* = 0.007) and current smokers (33% vs. 12%, *p* = 0.004), and these data should be taken into consideration, given the role of smoking in post-operative recurrence [82].

However, it is also important to point out that a score to evaluate the endoscopic recurrence in patients who underwent Kono-S anastomosis does not exist, because the Rutgeerts score is not standardized for the use in this setting.

#### 3.4.3. Mesenteric Excision

Given the possible role of the mesentery in the pathogenesis of recurrence, it has been suggested that mesenteric excision during ICR could be a protective factor toward recurrence.

Coffey et al. conducted a study where patients underwent ICR with conventional mesentery-sparing resection (*n* = 30) or ICR plus excision of the mesentery (*n* = 34). The cumulative reoperation rates were 40% in the first group and 2.9% in the second group (*p* = 0.003). However, the length of follow up was shorter in the subjects undergoing ICR plus mesentery-excision and this may have underestimated the rate of recurrence. In a multivariable analysis, it was found that conventional mesentery-sparing resection was an independent predictor of surgical recurrence (*p* = 0.007) [25].

In a recent retrospective study including 126 patients with Crohn’s colitis, those who underwent colorectal resection with extensive mesenteric excision (*n* = 66) had a longer surgical POR-free survival time compared with patients who underwent colorectal resection with limited mesenteric excision (*p* = 0.01). The limited mesenteric excision group was an independent predictor of surgical POR (HR 2.67, 95% CI 1.04–6.85, *p* = 0.04) [26].

Despite these results, there is a need for RCTs to enable an objective assessment of extensive mesenteric resection to standardize how the surgeon should behave toward the mesentery during an ileo-cecal resection for CD. For this purpose, some randomized controlled trials are ongoing (NCT03172143, NCT04578392, NCT04538638, NCT03769922).

## 4. Strategies to Prevent POR

Ileo-cecal resection for CD is not curative and a large proportion of patients will suffer disease recurrence, as shown in the benchmark study conducted by Rutgeerts et al., in which recurrent endoscopic lesions were observed in 73% of the patients 1 year after surgery [83]. This high rate of endoscopic recurrence was also confirmed in a more recent study [84].

However, to date, the optimal strategy to prevent recurrence is a major topic of discussion, bearing in mind that approximately 20–30% of subjects may not experience endoscopic POR and that 40–50% will only exhibit intermediate lesions (Rutgeerts i1–i2), which carry a low risk of mid- and long-term clinical and surgical POR [11]. Currently, there are two accepted intervention strategies: initiation of early post-operative prophylactic therapy (systematic or based on the presence of risk factors for recurrence) or initiation of therapy based on endoscopic evidence of disease recurrence at colonoscopy performed 6–12 months after ICR (endoscopy-driven therapy) [11].

The European Crohn’s and Colitis Organisation (ECCO) guidelines [12] and the British Society of Gastroenterology (BSG) [14] recommend prophylactic treatment in high-risk patients. In contrast, the American Gastroenterology Association (AGA) suggests early pharmacological prophylaxis in all patients, regardless of the presence of risk factors [13]. However, there is disagreement among international guidelines when it comes to defining an individual as being at high risk of recurrence: the BSG requires the presence of at least two risk factors, while the AGA and the ECCO require the presence of a single risk factor [12,13,14] (Table 1).

Each strategy has advantages and disadvantages. The initiation of systematic prophylactic therapy yields good results in terms of medium- to long-term remission after intervention, but it is associated with a significant rate of overtreatment. The risk of drug-related side effects might overcome their potential preventive benefits in the subset of patients who would not have developed endoscopic or clinical POR [11]. Instead, starting a prophylactic therapy based on the patient’s risk of recurrence may avoid over- and undertreatment, but this strategy is complicated by the difficulty of categorizing patients into those at high and low risk according to the presence or absence of risk factors. Studies conducted after the publication of these international guidelines have not resolved the debate on the best management of POR. Supporting the BSG guidelines, Joustra et al. did not observe a significant association between endoscopic POR and a high-/low-risk profile. Only patients with a combination of any three or more risk factors showed increased odds of developing endoscopic recurrence [29]. In another retrospective study of 376 operated CD patients, although prophylactic therapy decreased the rate of endoscopic POR within 1 year in high-risk patients compared to endoscopy-driven therapy (HR 0.48, *p* = 0.04, NNT = 5), there was no significant difference in clinical recurrence within 3 years between the two strategies (HR 1.06, *p* = 0.82, NNT 22). Interestingly, a wide numerical difference in the rate of clinical POR at 3 years was seen in a sub-cohort of patients with ≥3 ECCO-defined risk factors compared to patients managed with endoscopy-driven therapy (28.6% vs. 62.5%; *p* = 0.11, respectively), suggesting a cumulative impact associated with the presence of multiple risk factors [85]. Again, in the retrospective study by Dragoni et al., in CD operated patients with only one risk factor for POR, immediate prophylaxis did not significantly decrease the rate of endoscopic recurrence (prophylaxis group 36.1% vs. endoscopic-driven approach 45.5%; *p* = 0.10) or severe endoscopic recurrence (prophylaxis group 9.8% vs. endoscopic-driven approach 15.7%; *p* = 0.15) within 12 months after surgery [86]. On the other hand, a recent prospective cohort study of 213 CD patients undergoing ICR showed that clinical risk stratification (high-risk patients if ≥1 risk factor: active smoking, penetrating disease, prior ICR) had an acceptable predictive value in terms of endoscopic recurrence (Rutgeerts score ≥ i2b) at 6 months. The endoscopic POR was higher in patients treated without prophylaxis than with prophylaxis in both low-risk patients (45% vs. 16%, *p* = 0.012) and high-risk patients (49% vs. 26%, *p* = 0.019) [67]. D’Amico et al. retrospectively analyzed 141 patients who underwent surgery for CD and observed that a higher rate of endoscopy recurrence was detected in patients without prophylaxis therapy compared with those treated (80.8% vs. 45.2%, *p* < 0.0001) and that the absence of biologic therapy was independently associated with the risk of endoscopic POR in the logistic regression model (OR 0.22, 95% CI 0.1–0.51; *p* = 0.0004). Furthermore, according to the Kaplan–Meier analysis, patients without prophylaxis therapy at baseline had a >23.3% 5-year rate of hospitalization and surgery (log-rank *p* = 0.0221) and a >49.7% 5-year rate of medical therapy escalation (log-rank *p* = 0.0013) in contrast with subjects treated with immediate prophylaxis [87]. In the retrospective PORCSE study, the endoscopic POR rate was significantly higher in patients treated with endoscopy-driven therapy strategy compared to patients receiving early prophylaxis therapy (53.8% vs. 41.5%; OR 1.81, *p* = 0.039, respectively). No significant difference was observed in severe endoscopic POR (OR 1.29, *p* = 0.517). However, individuals in the endoscopy-driven therapy group exhibited higher clinical POR rates (35.7% vs. 17.7%; OR 3.05, *p* = 0.002, respectively) and higher surgical recurrence rates (13.2% vs. 6.7%; OR 2.59, *p* = 0.051, respectively) [88].

## 5. Medical Therapy to Prevent POR

Given the high rates of endoscopic, clinical, and surgical recurrence after intestinal resection for CD, there is a clear need to identify the best prophylactic therapy to reduce the risk of recurrence.

For this endpoint, a plethora of nonbiologic therapies and biological drugs have been assessed (Table 2).

### 5.1. Corticosteroids

Only two studies, conducted before the introduction of biological therapy, evaluated the role of budesonide in preventing POR.

In a multicenter randomized double-blind placebo-controlled trial lasting 1 year, 83 patients were divided into 2 groups (*n* = 43 budesonide 3 mg daily, *n* = 40 placebo). The recurrence rate after 1 year (endoscopic and/or clinical) was 57% in the budesonide group and 70% in the placebo group, without a statistically significant difference [89]. Similar results were described in another study by Hellers et al. In this study, unlike the previous one, the budesonide group took a dosage of 6 mg daily. Oral budesonide did not provide any benefit in preventing endoscopic recurrence, both at 3 months (budesonide 31% vs. placebo 35%) and at 12 months (52% vs. 58%, respectively) [90]. Regarding systemic corticosteroids, to the best of our knowledge, no papers have been published.

However, despite the lack of scientific evidence for this therapeutic intervention, in clinical practice corticosteroids are applied in approximately one-third of patients after ileocolonic resection [110].

### 5.2. Mesalazine

The role of mesalamine in preventing POR has been assessed in previous studies, mostly between the 1990s and 2000s.

Three studies demonstrated a positive role of mesalazine in preventing POR. In the RCT study conducted by Caprilli et al., mesalazine at a dosage of 2.4 g/day was effective compared to the placebo in preventing POR over a 2-year period, preventing 39% of all recurrences and 55% of the severe recurrences [111]. Similarly, the dosage of 3.0 gr/day also significantly decreased the risk of POR in two other RCTs [112,113]. Furthermore, in the study by Brignola et al., mesalazine was also useful in reducing the severity of endoscopic recurrence [112].

Different results were reported in a subsequent placebo-controlled clinical trial (55 mesalazine vs. 51 placebo), where 3 gr/day mesalazine did not significantly reduce the rate of endoscopic recurrence at 12 weeks (mesalazine 50% vs. placebo 63%; *p* = 0.16) [94]. Negative results were observed in another randomized, placebo-controlled trial (*n* = 131 patients) assessing the efficacy of 6-mercaptopurine and mesalamine in the prevention of POR. Mesalamine (3 g/day) did not display a benefit over the placebo in terms of clinical recurrence (HR 0.62; *p* = 0.123) and endoscopic recurrence (HR 1.10; *p* = 0.82) over 2 years [95].

Lochs et al. investigated a slow-release form of mesalamine (4 gr/day) with a significant release of the drug in the small bowel (Pentasa; Ferring A/S, Vanløse, Denmark), which could provide a potential advantage due the fact that it is the most frequent location of POR. In this placebo-controlled RCT (152 mesalamine vs. 166 placebo), the cumulative relapse rate after 18 months was not significantly different in the 2 groups (Pentasa 24.5% vs. placebo 31.4%; *p* = 0.10). However, it was showed a significant reduction in the relapse rate in the subgroup of patients with isolated small bowel disease (Pentasa 21.8% vs. placebo 39.7%; *p* = 0.02) [96]. Interestingly, some authors demonstrated that the mucosa concentration of mesalamine in the peri-anastomotic mucosa was significantly lower in subjects with POR, suggesting a correlation between the mucosa mesalazine concentration and its clinical effectiveness [114]. Despite this finding, in the RCT conducted by Caprilli et al. assessing the relationship between dosage and response (101 pts with mesalazine 4.0 g/day vs. 105 pts with mesalazine 2.4 g/day), a higher efficacy of the 4.0 g/day regimen was not observed over the 2.4 g/day regimen in preventing endoscopic and clinical POR at 1 year of follow up [97].

Finally, in a recent meta-analysis from the Cochrane Library, including data from RCTs, during a follow-up period of 12 to 72 months, 36% (131/361) of 5-ASA patients relapsed (clinical relapse) compared to 43% (160/369) of placebo participants (RR 0.83, 95% CI 0.72 to 0.96. I2 = 0%). However, for the prevention of endoscopic POR, the evidence was uncertain [98].

### 5.3. Antibiotics and Probiotics

Given the established role of the microbiota, it has been postulated that its modulation with antibiotics or probiotics may have a role in the management of POR.

The class of antibiotics most evaluated is nitroimidazoles, particularly metronidazole, due to its spectrum of action against anaerobic bacteria. In a placebo-controlled RCT, ICR patients were randomized to receive metronidazole (20 mg/kg daily, *n* = 30) versus placebo (*n* = 30) for 3 months. At 12 weeks, 75% of patients in the placebo arm had recurrent lesions in the neo-terminal ileum compared to 52% patients in the metronidazole arm (*p* = 0.09). The incidence of severe endoscopic recurrence was significantly reduced by metronidazole (13%) compared to placebo (43%; *p* = 0.02). Patients in the metronidazole group experienced more frequent side effects: seven patients in this group discontinued therapy compared to two patients in the placebo group. The effects of this intervention appeared to be prolonged, with a reduction in clinical recurrence rates at 1, 2, and 3 years. In fact, even short-term treatment (3 months) may offer a long-term benefit [91]. Similar results were obtained in another placebo-controlled RCT, where ornidazole 1 gr/day (*n* = 40) versus placebo (*n* = 40) was evaluated after 1 year of therapy. Ornidazole significantly reduced both clinical recurrence (7.9% vs. 37.5%, respectively; *p* = 0.0046) and endoscopic recurrence (53.6% vs. 79%, respectively; *p* = 0.037). Also, in this study, adverse events were more common in the antibiotic treated group [92]. Instead, in a pilot randomized, placebo-controlled trial, ciprofloxacin (500 mg bid) did not demonstrate a lower rate of endoscopic recurrence with a higher rate of drug discontinuation due to drug-associated adverse events [93]. With regard to probiotics, several mixtures have been assessed in RCTs, but none demonstrated efficacy in preventing POR [115,116,117,118].

### 5.4. Immunomodulators

Various RCTs have evaluated the effectiveness of thiopurines in preventing POR.

One randomized, placebo-controlled, double-blind trial (*n* = 131) assessing the efficacy of 6-mercaptopurine (6-MP) and mesalamine in the prevention of POR demonstrated that 6-mercaptopurine (50 mg/day) was more effective than the placebo (*p* < 0.05) in preventing clinical recurrence (50% vs. 77%, respectively) and endoscopic recurrence (43% vs. 64%, respectively) over 2 years [95].

On the other hand, an interesting randomized (1:1, *n* = 240), placebo-controlled, double-blind trial demonstrated that 6-mercaptopurine (1 mg/kg) was not effective compared to the placebo in preventing clinical recurrence in all patients (13% vs. 23%, respectively, HR 0.54; 95% CI 0.27–1.06), only in a specific subgroup, namely smokers. In the smoker subgroup, 6-mercaptopurine achieved a lower rate of clinical recurrence compared to the placebo (10% vs. 46%, respectively, HR 0.13; 95% CI 0.04–0.46) [99].

Regarding azathioprine (AZA), positive results in the prevention of POR were observed in the RCT conducted by D’Haens et al., which included operated CD patients (*n* = 81) with one or more risk factors for recurrence. In this study, azathioprine for 12 months plus metronidazole for 3 months was superior to metronidazole for 3 months alone in reducing endoscopic POR at 12 months (43.7% vs. 69.0%; *p* = 0.048) [100].

On the other hand, a subsequent RCT of 50 patients showed that azathioprine for 1 year plus metronidazole did not significantly reduce the risk of endoscopic recurrence compared to azathioprine alone at 1 year of follow up (36% vs. 56%; *p* = 0.15). Based on the results of this pilot study, it can be speculated that the addition of a short initial course of metronidazole alongside long-term azathioprine does not significantly reduce the chances of early endoscopic recurrence and that azathioprine may play the major role in reducing POR in the combination therapy of azathioprine plus metronidazole [101].

Furthermore, in the randomized study conducted by Reinisch et al., azathioprine demonstrated superiority compared to mesalazine for the prevention of CD post-operative clinical recurrence in patients with endoscopic recurrence (0% vs. 10.8%, respectively, *p* = 0.031). Additionally, the median improvement in the Rutgeerts score was significantly greater in the azathioprine group [102]. Finally, a recent Cochrane meta-analysis (*n* = 408 participants) stated that thiopurines were superior to the placebo in preventing clinical POR after 12–36 months of follow up (51% vs. 64%, RR 0.79, 95% CI 0.67–0.92) [103].

### 5.5. Anti-TNF

Regarding the preventive role of infliximab, a pivotal prospective, small-sample-size, non-randomized study by Sorrentino et al. showed that the combination of infliximab and low-dose oral methotrexate (*n* = 7) was more effective in preventing POR for 2 years compared to the control group (*n* = 16) treated with mesalamine 2.4 g/day (0% vs. 75%, respectively) [119]. In a follow up study, Sorrentino et al. re-treated 10 operated CD patients who developed endoscopic recurrence after discontinuing infliximab. Re-treatment with lower doses of infliximab (3 mg/kg, every 8 weeks) restored and maintained endoscopic remission for 1 year in all the patients [120].

Even studies with a higher quality of evidence confirmed these findings. A 3-year prospective, randomized, open trial conducted by Yoshida et al. demonstrated that early intervention (4 weeks after surgery) with infliximab (*n* = 15) prevented clinical recurrence at 36 months (remission rate 93.3% vs. 56.3%; *p* < 0.03) and endoscopic recurrence at 12 months (remission rate 78.6% vs. 18.8%; *p* = 0.004) compared to the control group (*n* = 16) [121]. Again, in a pair-matched study comparing 100 patients who had received post-operative infliximab with those who had not, the multivariate analysis showed that post-operative infliximab maintenance therapy was the only significant factor preventing surgical recurrence (HR 0.08, 95% CI 0.01–0.53; *p* = 0.03) [122]. Similar results were observed in the PREVENT trial, which was the first extensive, multicenter, placebo-controlled study assessing the efficacy of infliximab in preventing post-operative recurrence of CD. A total of 297 patients, randomly assigned (1:1) to infliximab or placebo, were included. At week 76, endoscopic recurrence was 30.6% in the infliximab group compared to 60.0% in the placebo group. The absolute risk reduction (ARR) with infliximab was 29.4% (95% CI 18.6–40.2%; *p* < 0.001). However, regarding clinical recurrence, a smaller proportion of patients in the infliximab group had POR compared with the placebo group, but this difference was not statistically significant (12.9% vs. 20.0%; ARR with infliximab 7.1%; 95% CI 1.3–15.5%; *p* = 0.097) [104]. Subsequently, also in an RCT by Fukushima et al., despite the small sample size (*n* = 38), at 2 years, 94.7% of patients in the placebo group had experienced a recurrence of disease (clinical and/or endoscopic) compared to 52.6% in the infliximab group (*p* = 0.0032) [105]. Also, a recent meta-analysis (455 patients, 7 trials) confirmed the efficacy of infliximab in preventing POR. Overall, infliximab significantly reduced the rates of endoscopic recurrence (RR = 0.421, 95% CI 0.328–0.539; *p* < 0.001) and clinical recurrence (RR = 0.519, 95% CI 0.349–0.774; *p* = 0.001) [106].

Concerning adalimumab, the first pilot study assessing the efficacy of adalimumab in preventing POR was a small-sample-size, prospective study, which included 8 “high-risk” patients who started adalimumab 14 days after surgery. At 24 months of follow up, 6 of 8 patients (75%) did not have clinical and endoscopic POR [123]. Similarly, another multicenter, prospective, observational study including 29 patients with two or more risk factors for POR observed that after starting prophylaxis therapy with adalimumab, only 13.7% of the patients developed clinical recurrence and only 20.7% developed endoscopic recurrence after 1 year [124]. Homogeneous results were observed in subsequent observational Japanese studies [125,126]. Despite these findings, no placebo-controlled RCTs assessing adalimumab in terms of POR prevention have been performed.

Finally, there seems to be no significant difference in the effectiveness of preventing POR between infliximab and adalimumab. In the open-label, prospective study conducted by Tursi et al., infliximab and adalimumab were similar in preventing both histological (30% vs. 20%, respectively; *p* = 1.0) and endoscopic (20% vs. 10%, respectively; *p* = 1.0) recurrence [127].

Also, the comparative retrospective study by Preda et al. observed that the rate of endoscopic recurrence at 12 months was similar between the two anti-TNF agents (IFX 29% vs. ADA 33%) [128]. Several other studies and meta-analysis confirmed the equivalence of both anti-TNF agents in preventing POR [129,130,131,132,133].

### 5.6. Anti-TNF versus Azathioprine

Regarding the comparison between infliximab and azathioprine in terms of preventing POR, few data are available. In a prospective, randomized, small-sample-size study including 22 operated CD patients with a “high-risk” of recurrence, the incidence of endoscopic and clinical POR at 1 year did not differ significantly between the 11 patients receiving infliximab and the 11 patients receiving azathioprine. However, infliximab was more effective than azathioprine in reducing histological activity (18% vs. 80%, respectively; *p* = 0.008) [107].

For adalimumab, Savarino et al. performed an RCT (*n* = 51) comparing adalimumab, azathioprine, and mesalamine in preventing POR. The rate of endoscopic POR was lower in the adalimumab group (6.3%) compared with the AZA (64.7%) (OR 0.036; 95% CI 0.004–0.347) and mesalamine groups (83.3%) (OR = 0.013; 95% CI 0.001–0.143). Similarly, there was a lower proportion of subjects in clinical recurrence in the adalimumab group (12.5%) compared with the AZA (64.7%) (OR = 0.078; 95% CI 0.013–0.464) and mesalamine groups (50%) (OR = 0.143; 95% CI 0.025–0.819) [108]. In a subsequent analysis of the POCER study, 101 operated patients with risk factors for POR (smoker, perforating disease, ≥2nd operation) were treated with either AZA/6-MP (*n* = 73) or adalimumab (*n* = 28) in thiopurine-intolerant patients. Endoscopic POR occurred in 45% in the thiopurine group vs. 21% in the adalimumab group (*p* = 0.028). Interestingly, complete mucosal normality (Rutgeerts i0) only occurred in 23% in the thiopurine group vs. 54% in the adalimumab group (*p* = 0.003) [134]. On the contrary, in a subsequent RCT, although treatment with adalimumab failed less than azathioprine at week 52, the former drug did not demonstrate superiority over azathioprine for prophylaxis of CD recurrence in a unselected population for POR risk factors (42.2% vs. 59%, *p* = 0.12). Interestingly, tolerance of adalimumab was better than that of AZA (discontinuation in ADA 4.4% vs. discontinuation in AZA 23.2%; *p* = 0.011) [109]. A recent meta-analysis (6 studies, 645 patients) observed that anti-TNF-α agents are superior to thiopurine in preventing endoscopic and clinical POR. The relative risk (RR) was 0.52 (95% CI, 0.33–0.80) for endoscopic recurrence, 0.50 (95% CI, 0.26–0.96) for clinical recurrence and 0.41 (95% CI, 0.21–0.79) for severe endoscopic recurrence. Interestingly, the advantage of anti-TNF-α agents over thiopurines was observed in both low- and high-risk individuals. In particular, the absolute risk difference for endoscopic recurrence at 1 year of follow up was 23.3% in low-risk patients, while it was 39.9% in high-risk patients [44].

### 5.7. Ustekinumab and Vedolizumab

The data regarding the effectiveness of ustekinumab (UST) and vedolizumab (VDZ) in preventing POR are very limited and only one RCT is available.

Regarding vedolizumab, in a retrospective cohort comparing the risk of POR between individuals receiving vedolizumab and anti-TNFα agents, the rate of endoscopic remission at 6–12 months in the vedolizumab group was significantly lower than in the anti-TNF-α agent group (25% vs. 66%, respectively; *p* = 0.01). However, the rates of clinical and biological remission were similar in both groups. Importantly, vedolizumab was identified as the only factor associated with an increased risk of endoscopic POR in both univariate and multivariate analyses [135].

The preliminary results of the REPREVIO trial, a prospective placebo-controlled RCT investigating the efficacy of vedolizumab in preventing POR in patients with at least one risk factor, were recently shown: endoscopic remission was observed in 18/43 patients on vedolizumab versus 1/37 on the placebo (42% vs. 3%, *p* < 0.001, respectively) and patients in the treatment group had a 77.8% (95% CI 66.4–86.29%) chance of having a better Rutgeerts score than the control patients (*p* < 0.0001) [136].

Regarding ustekinumab, in a comparative, small-sample-size study by Buisson et al., the effectiveness of ustekinumab (*n* = 32) was compared to azathioprine (*n* = 31) in preventing POR. After adjusting according to the propensity score analysis for the main risk factors and for the use of thiopurines or ustekinumab prior to surgery, the rate of endoscopic POR at 6 months was lower in patients treated with ustekinumab compared to azathioprine (28% vs. 54.5%, *p* = 0.029). It is noteworthy that this result was largely driven by moderate disease (Rutgeerts score i2) as no significant difference was observed when the Rutgeerts score was ≥i3 (16.9% vs. 27.9%, *p* = 0.24) [137].

In a real-world, retrospective, multicenter study conducted by Yanai et al., 297 patients underwent POR prophylaxis biologic treatment (anti TNF = 224, VDZ = 39, UST = 34). The endoscopic POR at 1 year was 61.8% in the ustekinumab group, 33% in the vedolizumab group, and 40.2% in the anti-TNF group. However, the patients treated with ustekinumab or vedolizumab were more biologically experienced, with higher rates of previous surgery. Indeed, after inverse probability treatment weighting, the risk of endoscopic POR within 1 year between ustekinumab or vedolizumab and the anti-TNF was comparable (UST vs. anti-TNF: OR 1.86, 95% CI 0.79–4.38. VDZ vs. anti-TNF: OR 0.55, 95% CI 0.25–1.19) [138].

Also, a more recent retrospective, cohort, multicenter study analyzed 278 patients who received post-operative prophylaxis with different biologic agents (anti-TNF = 223, VDZ = 27, UST = 28). Contrary to the previous study, after adjusting for factors associated with POR, only the initiation of an anti-TNF agent within 4 weeks was associated with a reduction in POR (anti-TNF: aHR 0.61, 95% CI 0.40–0.93. VDZ: aHR 1.44, 95%CI 0.59–3.56. UST: aHR 2.06, 95% CI 0.84–5.06) [139].

Directly comparing vedolizumab vs. ustekinumab in preventing POR, only a very recent prospective study assessed this topic (*n* = 40 treated with UST and *n* = 25 treated with VDZ). The cumulative probability of clinical POR at 12 months was 32% for ustekinumab and 30% for vedolizumab. The rate of endoscopic POR at 18 months was 42% for the ustekinumab group and 40% for the vedolizumab group [140].

Further investigations, including randomized, controlled trials, are needed to establish the utility of vedolizumab and ustekinumab in preventing post-operative recurrence of Crohn’s disease.

### 5.8. The Use of Anti-TNF despite Its Use before Surgery

It could be hypothesized that if a prior anti-TNF treatment had proven ineffective before surgery, the efficacy of the anti-TNF agents in preventing POR might be diminished.

In a retrospective analysis of operated patients (*n* = 57) treated with anti-TNF (initiated within 3 months of surgery), 37% and 42% patients had a previous exposure to 1 and ≥1 anti-TNF agents, respectively. The cumulative rates of post-operative endoscopic recurrence at 2 years were 45.5% in patients exposed to ≥2 anti-TNFα as compared with 29.1% in patients exposed to ≤1 anti-TNFα before surgery (*p* = 0.07). Multivariable analysis identified previous exposure to ≥2 anti-TNFα agents a as risk factor for clinical and/or endoscopic POR (HR = 4.2; 95% CI: 1.8–10.2, *p* = 0.001) [141].

However, different data were observed by Assa et al. In this retrospective study on pediatric patients, the same anti-TNF used before surgery was prescribed as prophylaxis in 18 patients and compared with 35 controls (patients never exposed to anti-TNF). Interestingly, similar proportions of patients from both groups were in clinical remission at the end of the follow up (1.8 years, interquartile range, 1–2.9 years): 83% vs. 80% for pre-surgery anti-TNF failure and controls, respectively (*p* = 0.8). Furthermore, no significant differences in endoscopic remission were observed at 12 months between the two groups (50% vs. 45%, respectively; *p* = 0.7) [142].

Similarly, in a recent retrospective, multicenter study, anti-TNF treatment remained an effective option to prevent POR in operated patients with preoperative anti-TNF failure. Among the 119 subjects included, 71 received anti-TNF and 48 another treatment (18 UST/VDZ, 20 AZA and 3 MTX) for POR prophylaxis. The rate of endoscopic/radiological recurrence at 2 years was 23.9% in the anti-TNF group compared with 44.9% in the other group (*p* = 0.011) [143]. Also, in a subgroup analysis of an above-mentioned meta-analysis, anti-TNF agents remained superior to azathioprine in patients with pre-surgery exposure to anti-TNF [44]. One of the hypotheses for the efficacy of anti-TNF even in patients already exposed to anti-TNF before surgery is that in severely inflamed tissue with a high inflammatory burden, tissue injury and local hypoxia might limit drug penetrance to its target. In these cases, intestinal resection may restore the efficacy of traditional biologic therapy such as anti-TNF agents [142]. Indeed, Zorzi et al. observed that TNF-a was not increased in CD patients with established lesions and a long history of disease requiring bowel resection. Conversely, elevated expression of TNF-a was seen in the unaffected neo-terminal ileum after surgery, playing a pathogenic role in CD POR [23].

This could explain the role and efficacy of anti-TNF agents in preventing POR regardless of the pre-surgery exposition status.

## 6. Discussion

The management of post-operative CD is challenging. Ensuring timely, effective, and safe prevention of recurrence is essential but difficult. The risk of overtreatment lies in exposing patients to undesired adverse events, along with the long-term risks and costs of associated medications. On the other hand, undertreatment may lead to missed opportunities to prevent bowel damage and complications, and to the necessity of additional surgery [144]. To balance the aggressive strategy of immediate systematic medical prophylaxis with the more conservative strategy of endoscopy-guided therapy, previous research has shown that a subset of patients has specific risk factors that place them at increased risk of early disease recurrence [144].

Therefore, the scope of this new strategy is to stratify the patients after ICR and to detect the subset of individuals at high risk of recurrence in whom the benefit of early systematic prophylaxis outweighs the risk of side effects [11,145].

Despite the low quality of the evidence, most international guidelines recommend this “risk stratification strategy” (weak recommendation), in which early medical prophylaxis is started in only high-risk patients. However, the definition of a high-risk patient is not homogeneous between guidelines (at least one risk factor vs. >one risk factors) and the current approach to risk stratification in recurrence demonstrated limited effectiveness in recent real-world studies, potentially resulting in many individuals receiving biologic therapy with uncertain concrete benefit [12,13,14,144]. Indeed, despite the retrospective design of most studies published after these guidelines, the majority of them confirmed the need for multiple risk factors in order to achieve benefit from prophylaxis therapy [11].

In particular, the weight of only one risk factor seems insufficient to gain a significant decrease in the POR rate with systematic prophylaxis. The ongoing prospective, randomized SOPRANO-CD study (NCT05169593) will evaluate if the endoscopy-driven therapy strategy is as efficacious as systematic prophylactic therapy in preventing endoscopic POR at week 86 in patients undergoing ICR with at least one clinical risk factor.

In the near future, when the weight of all the risk factors will be clearer (also the histological ones), the concept of a cumulative effect due the presence of multiple risk factors and the right number of risk factors needed to define a high-risk patient will help the physician in determining the most accurate threshold for initiating prophylactic therapy with an optimal risk–benefit ratio [11,85,144]. Furthermore, an additional tool in the management of post-operative CD may come from the evidence on the potential role of biomarkers, in particular fecal calprotectin, as early predictors of disease recurrence [146,147].

**Figure 2:**
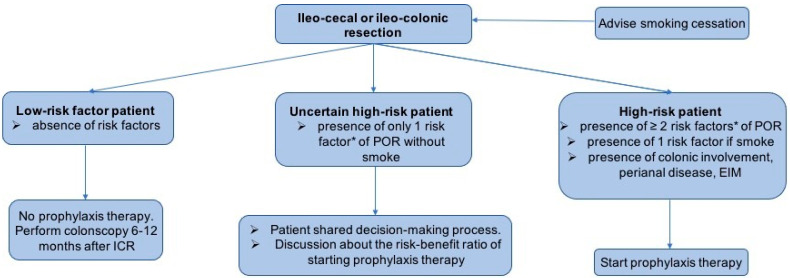
Possible strategies to prevent post-operative recurrence. * risk factors: smoking, prior intestinal surgery, penetrating disease at index surgery, granulomas in resection specimen, myenteric plexitis, <30 years old patient, extensive (>50 cm) bowel disease (adapted from ECCO, AGA, and BSG guidelines).

Based on the existing data, we suggest the following treatment algorithm after ICR (Figure 2), keeping in mind that the quality of the evidence consists of retrospective studies. This algorithm seeks to stratify patients by integrating the known risk factors for POR and also including clinical features that demand the continuation (or introduction) of biologic therapy (i.e., prior colonic involvement, active perianal disease, concomitant extraintestinal manifestations) and a patient-shared decision-making process [11].

If prophylactic therapy is required, the current data suggest preferring anti-TNF as the first-line agent, even in patients already exposed to this class of drugs before surgery. However, the research in the field of IBD therapy is rapidly growing. In the future, well-designed studies (including patients already exposed to anti-TNF or multiple biologics before surgery) are needed to assess the real efficacy of the more recent classes of drugs, including the new pipeline, such as selective IL-23p19 inhibitors and JAK inhibitors like upadacitinib.

## 7. Conclusions

Prevention of post-operative recurrence is essential to reduce the need for subsequent surgeries and potential bowel damage. Currently, treatment decisions primarily rely on clinical risk factor assessment, highlighting the importance of adopting more proactive prevention therapies for high-risk patients. However, additional research is necessary to establish evidence-based treatment protocols and validate tools for patient risk stratification, including different risk factors (i.e., histological, surgical). When needed, biologic agents have demonstrated effectiveness in reducing recurrence, although comparative studies are still limited. While anti-TNF agents show promising results, other biological medications warrant further investigation. Advances in medical and preventive strategies hold the promise of significantly improving post-operative outcomes in Crohn’s disease.

## Figures and Tables

**Table 1:** Different strategies between international guidelines.

	Risk Factors Considered	Number of Risk Factors to Define a High-Risk Patient	When to Start Prophylactic Therapy	Recommended Therapy
ECCO, 2017 [12]	- Current smoking - Prior intestinal surgery - Penetrating disease at index surgery - Perianal location - Granulomas in resection specimen - Myenteric plexitis	1	At least 1 risk factor	Thiopurines or anti-TNFs High-dose mesalamine is an option after an isolated ileal resection
AGA, 2017 [13]	- Age ≤ 30 years - Active smoking - ≥2 prior surgeries for penetrating disease, with or without perianal disease	1	Start systematically	Thiopurines or anti-TNFs
BSG, 2019 [14]	- Active smoking - Penetrating disease - Multiple resections - Perianal fistulae - Extensive small bowel disease (≥50 cm ileum) - Residual active disease - Granulomas or myenteric plexitis	2	At least 2 risk factors	Thiopurines or anti-TNFs

**Table 2:** RCTs and meta-analysis assessing the effectiveness of pharmacological interventions for the prevention of post-operative recurrence.

**CORTICOSTEROIDS**			
**Author and Year of Publication**	**Number of Patients Included**	**Drug Investigated**	**Results**
Ewe et al., 1999 [89]	RCT (*n* = 83)	Budesonide 3 mg/day versus placebo	Similar recurrence rate (endoscopic and/or clinical) (57% vs. 70%, *p* = ns) at 1 year
Hellers et al., 1999 [90]	RCT (*n* = 129)	Budesonide 6 mg/day versus placebo	Similar endoscopic POR at month 3 (31% vs. 52%, *p* = ns) and at month 12 (52% vs. 58%, *p* = ns)
ANTIBIOTICS			
**Author and Year of Publication**	**Number of Patients Included**	**Drug Investigated**	**Results**
Rutgeerts et al., 1995 [91]	RCT (*n* = 60)	Metronidazole (20 mg/kg daily) versus placebo for 3 months	Reduced endoscopic (52% vs. 75%, *p* = 0.09), severe endoscopic (13% vs. 43%, *p* = 0.02) at month 3 and reduced clinical recurrence at 1 year (4% vs. 25%, *p* = 0.044)
Rutgeerts et al., 2005 [92]	RCT (*n* = 80)	Ornidazole (1 gr/day) versus placebo for 1 year	Reduced both clinical (7.9% vs. 37.5%; *p* = 0.0046) and endoscopic recurrence (53.6% vs. 79%; *p* = 0.037) at 1 year
Herfarth et al., 2013 [93]	RCT (*n* = 33)	Ciprofloxacin (500 mg/twice daily) versus placebo for 6 months	Similar endoscopic (65% vs. 69%, *p* < 0.805) POR at 6 months
MESALAZINE			
**Author and Year of Publication**	**Number of Patients Included**	**Drug Investigated**	**Results**
Florent et al., 1996 [94]	RCT (*n* = 126)	Mesalazine (3 gr/day) versus placebo for 3 months	Similar endoscopic recurrence at 12 weeks (50% vs. 63%; *p* = 0.16)
Hanauer et al., 2004 [95]	RCT (*n* = 131)	6-MP (50 mg/day) versus mesalazine (3 gr/day) versus placebo for 24 months	No statistically significant difference between mesalazine and placebo in terms of clinical recurrence (HR 0.62; *p* = 0.123) and endoscopic recurrence (HR 1.10; *p* = 0.82) over 2 years
Lochs et al., 2000 [96]	RCT (*n* = 318)	Pentasa (4 gr/day) versus placebo for 18 months	Similar cumulative relapse rates (24.5% vs. 31.4%; *p* = 0.10) at 18 months
Caprilli et al., 2003 [97]	RCT (*n* = 206)	Mesalazine: 4 gr/day versus 2.4 gr/day for 1 year	No statistically significant differences between endoscopic recurrence (score > 1) (33% vs. 43%; *p* = 0.19) and clinical (12% vs. 14%; *p* = 0.58) POR at 1 year
Gjuladin-Hellon et al., 2019 [98]	Meta-analysis (5 studies, *n* = 730)	Mesalazine versus placebo	During a follow-up period of 12 to 72 months, reduced clinical POR (36% vs. 43%, RR 0.83, 95% CI 0.72–0.96)
Gjuladin-Hellon et al., 2019 [98]	Meta-analysis (3 studies, *n* = 537)	Mesalazine versus placebo	During a follow-up period of 12 to 72 months, similar endoscopic POR (70% vs. 73%, RR 0.83, 95% CI 0.56–1.23)
IMMUNOMODULATORS			
**Author and Year of Publication**	**Number of Patients Included**	**Drug Investigated**	**Results**
Hanauer et al., 2004 [95]	RCT (*n* = 131)	6-MP (50 mg/day) versus mesalazine (3 gr/day) versus placebo for 24 months	6-MP (50 mg/day) was more effective than placebo (*p* < 0.05) in preventing clinical recurrence (50% vs. 77%, respectively) and endoscopic recurrence (43% vs. 64%, respectively) over 2 years
Mowat et al., 2016 [99]	RCT (*n* = 240)	6-MP (1 mg/kg) versus placebo for 3 years	Similar clinical recurrence (13% vs. 23%, HR 0.54; 95% CI 0.27–1.06), but in the smoker subgroup, 6-MP achieved lower rate of clinical POR (10% vs. 46%, HR 0.13; 95% CI 0.04–0.46)
D’Haens et al., 2008 [100]	RCT (*n* = 81)	AZA for 12 months + metronidazole for 3 months versus metronidazole alone for 3 months	The addition of AZA reduced endoscopic POR at 12 months (43.7% vs. 69.0%; *p* = 0.048)
Mañosa et al., 2013 [101]	RCT (*n* = 50)	AZA for 12 months + metronidazole for 3 months versus AZA alone for 12 months	No statistically significant difference with the addition of a course of metronidazole in terms of endoscopic POR both at 6 months (28% vs. 44%, *p* = 0.19) and at 12 months (36% and 56%, *p* = 0.15)
Reinisch et al., 2010 [102]	RCT (*n* = 78)	AZA (2.0–2.5 mg/kg/day) versus mesalazine (4 g/day) over 1 year	At 12 months, lower clinical recurrence for AZA (0% vs. 10.8%, *p* = 0.031) in patients with endoscopic recurrence. Additionally, greater median improvement in Rutgeerts score
Gjuladin-Hellon et al., 2019 [103]	Meta-analysis (3 studies, *n* = 408)	Thiopurines versus placebo	Thiopurines reduced clinical (51% vs. 64%, RR 0.79, 95% CI 0.67–0.92) POR after 12–36 months
ANTI-TNF			
**Author and Year of Publication**	**Number of Patients Included**	**Drug Investigated**	**Results**
Regueiro et al., 2016 [104]	RCT (*n* = 297)	Infliximab versus placebo	At week 76, reduction of endoscopic recurrence (30.6% vs. 60.0%, *p* < 0.001), but regarding clinical recurrence, no statistically significant difference (12.9% vs. 20.0%, *p* = 0.097)
Fukushima et al., 2018 [105]	RCT (*n* = 38)	Infliximab versus placebo	Lower recurrence rate (clinical and/or endoscopic) (52.6% vs. 94.7%, *p* = 0.0032) at 2 years
Huang et al., 2018 [106]	Meta-analysis (7 trials, *n* = 455)	Infliximab versus placebo	Reduced endoscopic (RR = 0.421, 95% CI 0.328–0.539; *p* < 0.001) and clinical POR (RR = 0.519, 95% CI 0.349–0.774; *p* = 0.001)
Armuzzi et al., 2013 [107]	RCT (*n* = 22)	Infliximab versus AZA	No difference in clinical and endoscopic POR, but clear reduction in histological activity (18% vs. 80%; *p* = 0.008) at 12 months
Savarino et al., 2013 [108]	RCT (*n* = 51)	ADA versus AZA versus mesalazine	Lower endoscopic POR in the ADA group (6.3%) compared with the AZA (64.7%) and mesalamine groups (83.3%). Similar data also for clinical recurrence
López-Sanromán et al., 2017 [109]	RCT (*n* = 84)	ADA versus AZA, both associated with metronidazole	ADA did not demonstrate superiority over AZA (42.2% vs. 59%, *p* = 0.12) at week 52
Beelen et al., 2022 [44]	Meta-analysis (6 trials, *n* = 645)	Anti-TNF versus thiopurine	Lower endoscopic and clinical POR with anti-TNF

Abbreviations: RCT: randomized controlled trial, CI: confidence interval, ns: non-significant, HR: hazard ratio, RR: relative risk, POR: post-operative recurrence, 6-MP: 6-mercaptopurine, AZA: azathioprine, TNF: tumor necrosis factor, ADA: adalimumab.

## Data Availability

Data sharing is not applicable. No new data were created or analyzed in this study.
